# Extremely robust photocurrent generation of titanium dioxide photoanodes bio-sensitized with recombinant microalgal light-harvesting proteins

**DOI:** 10.1038/s41598-019-39344-6

**Published:** 2019-02-14

**Authors:** Nina Lämmermann, Fabian Schmid-Michels, Aike Weißmann, Lutz Wobbe, Andreas Hütten, Olaf Kruse

**Affiliations:** 1 Bielefeld University, Faculty of Biology, Center for Biotechnology (CeBiTec), Universitätsstrasse 27, 33615 Bielefeld, Germany; 2 Bielefeld University, Department of Physics, Center for Spinelectronic Materials and Devices, Universitätsstrasse 25, 33615 Bielefeld, Germany

## Abstract

Bio-dyes for light harvesting in dye-sensitized solar cells (DSSC) have the advantage of being environmentally-friendly, non-toxic alternatives, which can be produced in a sustainable fashion. Free photosynthetic pigments are unstable in the presence of light and oxygen, a situation which can hardly be avoided during the operation of DSSCs, especially in large-scale applications. We therefore investigated the recombinant light-harvesting protein LHCBM6, which naturally occurs in the photosynthetic apparatus of the green microalga *Chlamydomonas reinhardtii* as a bio-dye in DSSCs. Photocurrent densities of up to 0.87 and 0.94 mA·cm^−2^ were determined for the DSSCs and solar energy to electricity conversion efficiencies (η) reached about 0.3% (100 mW·cm^−2^; AM 1.5 G filter applied). Importantly, we observed an unprecedented stability of LHCII-based DSSCs within long DSSC operation times of at least 7 days in continuous light and show that operation times are restricted by electrolyte decomposition rather than reduced dye performance, as could be demonstrated by DSSC reactivation following re-supplementation with fresh electrolyte. To the best of our knowledge, this is the first study analysing bio-dye sensitized DSSCs over such long periods, which revealed that during illumination an activation of the DSSCs occurs.

## Introduction

Oxygenic photosynthesis can be sub-divided into two major types of biochemical reactions. Light reactions provide energy (ATP) and reducing equivalents (NADPH) by the endergonic process of water-splitting, which is driven by light absorption in the photosynthetic antenna. The ATP and NADPH produced by the light reactions are required to fix inorganic carbon and to produce glucose within the Calvin-Benson cycle, a cycle of reactions which are collectively termed “dark reactions”. Green algae and higher plants absorb light energy using light-harvesting complexes (LHC) associated with both photosystems and spanning the thylakoid membrane. This light absorption provides the energy needed for charge separation in the reaction centres of photosystems I and II. Depending on their predominant location at PSI or PSII, pigment-binding LHC proteins are designated LHCI (LHCA) or LHCII and two main types of LHCII proteins can be distinguished. The most abundant major LHCII proteins (termed LHCBM in *C. reinhardtii*) form the peripheral antenna of PSII in green microalga and higher plants^1^, while the less abundant monomeric LHCII proteins (CP26/CP29 in *C. reinhardtii*) are located in close proximity to the PSII core complex. Excitation energy transfer towards the special pair chlorophyll of the PSII reaction centre (P680), whose excitation is followed by charge separation reactions and electron transfer to the electron carrier plastoquinone^2,3^, requires chlorophyll *a* and *b* as well as carotenoids bound to LHCII apoproteins. Besides being involved in excitation energy transfer, the carotenoids associated with LHCII (lutein, neoxanthin, and xanthophyll cycle pigments) are also implicated in dissipative processes (NPQ) and the scavenging of reactive oxygen species (ROS), when light energy is provided in excess^4,5^.

Dye-sensitized solar cells, have been introduced as a promising alternative to conventional solar cells by Grätzel and O’Reagan^6^. Typically, an organic dye is immobilized on the surface of wide bandgap semiconductor (e.g. mesoporous film of TiO_2_ nanoparticle), which upon excitation injects electrons into the conduction band of the semiconductor. The photoanode is connected to a counter electrode (typically translucent conductive oxide with thin platinum coating to act as a catalyst), where an electrolyte component (e.g. the I/I_3_^−^ redox couple) is reduced, while oxidation of the redox couple fills the electron gap created via excitation in the dye^7^. LHCII molecules isolated from spinach leaves and binding photosynthetic pigments have been used within DSSCs before^8–11^, but so far the use of recombinant LHCII proteins has not been tested. In the present study, we investigated whether recombinant LHCBM6 from the green microalga *Chlamydomonas reinhardtii* represents a suitable bio-dye in DSSC applications. This isoform has been shown to possess an enhanced quenching capacity *in vitro*^12^, which could be associated with an enhanced stability in DSSC applications. Furthermore, and in contrast to previous studies, we performed long-time measurements of LHCII-based DSSCs to characterize changes in the performance during operation.

## Results and Discussion

### Preparation of functional PSII-associated light-harvesting protein LHCBM6 by *in vitro* reconstitution

**Figure 1:**
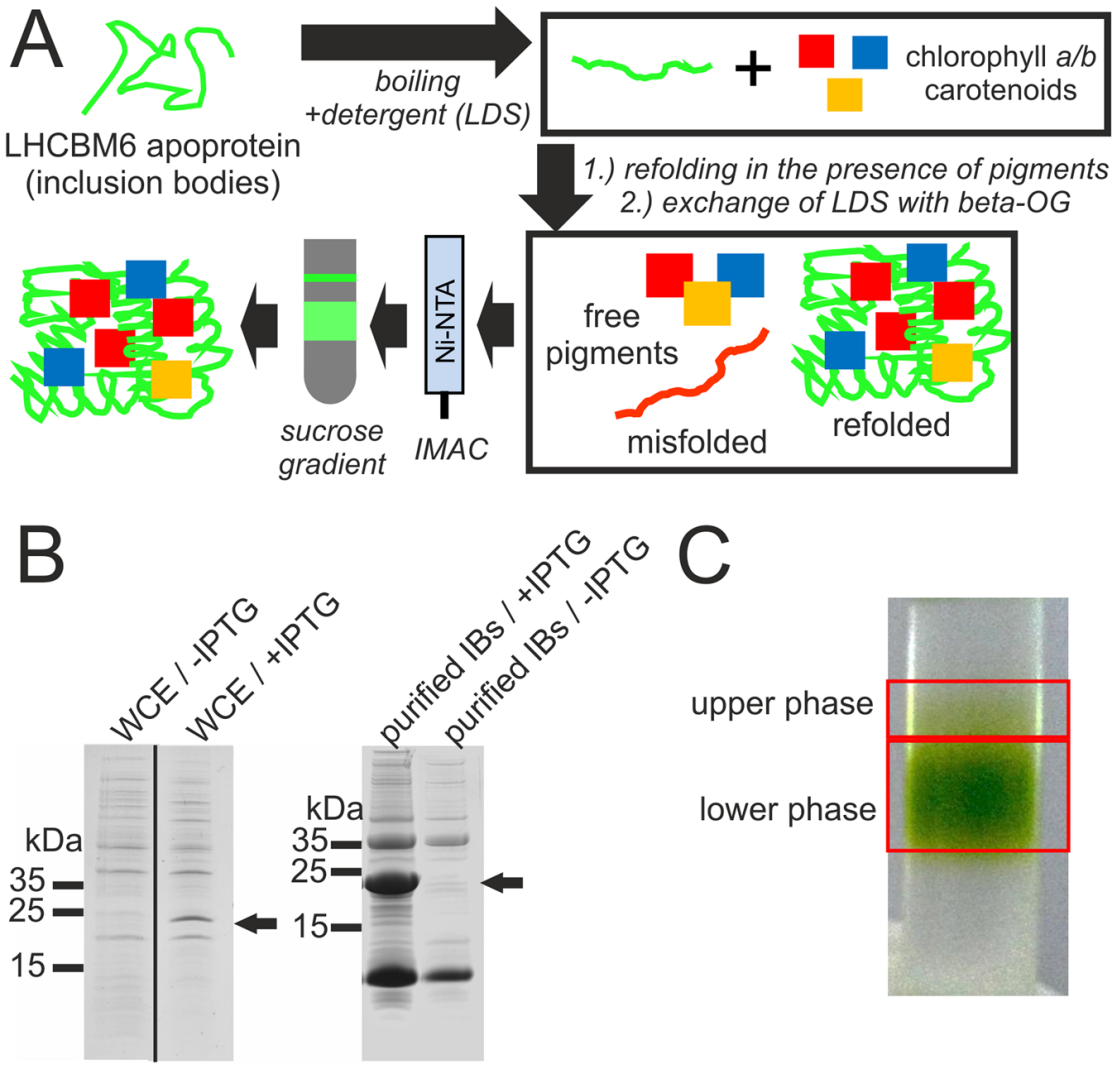
*In vitro* reconstitution of 6xHis-LHCBM6 from inclusion bodies after heterologous expression in *E. coli*. (**A**) Scheme depicting the workflow of *in vitro* reconstitution work. Misfolded and insoluble protein is resolubilized via boiling with the detergent LDS, resulting in unfolded protein. Refolding occurs during the exchange of LDS with the mild non-ionic detergent beta-OG and in the presence of pigments. Excess pigments are removed by IMAC and misfolded protein separated from the properly folded one by centrifugation through sucrose density gradient. Abbreviations: LDS/Lithium dodecyl sulphate; beta-OG/Octyl β-D-glucopyranoside**;** IMAC/immobilized metal affinity chromatography; Ni-NTA/Nickel nitrilotriacetic acid. (**B**) Left panel: Induction of His-LHCBM6 expression by addition (+) of Isopropyl-β-D-thiogalactopyranosid (IPTG) to cultures of *E. coli* cells harbouring the expression construct. Whole cell extracts (WCE) were separated by SDS-PAGE and stained with Coomassie Brilliant blue. Arrows indicate the band of His-LHCBM6. Right panel: Purification of inclusion bodies (IBs) from *E. coli* cultures either induced (+) or not induced (−) with IPTG. SDS-gels showing LHCBM6 production. left: in *E. coli* induced LHCBM6 expression, right: LHCBM6 purified from inclusion bodies. A black line indicates the grouping of gel lanes after cropping from original gels shown in the supplement. (**C**) Appearance of the sucrose gradient as the final step of *in vitro* reconstitution.

**Figure 2:**
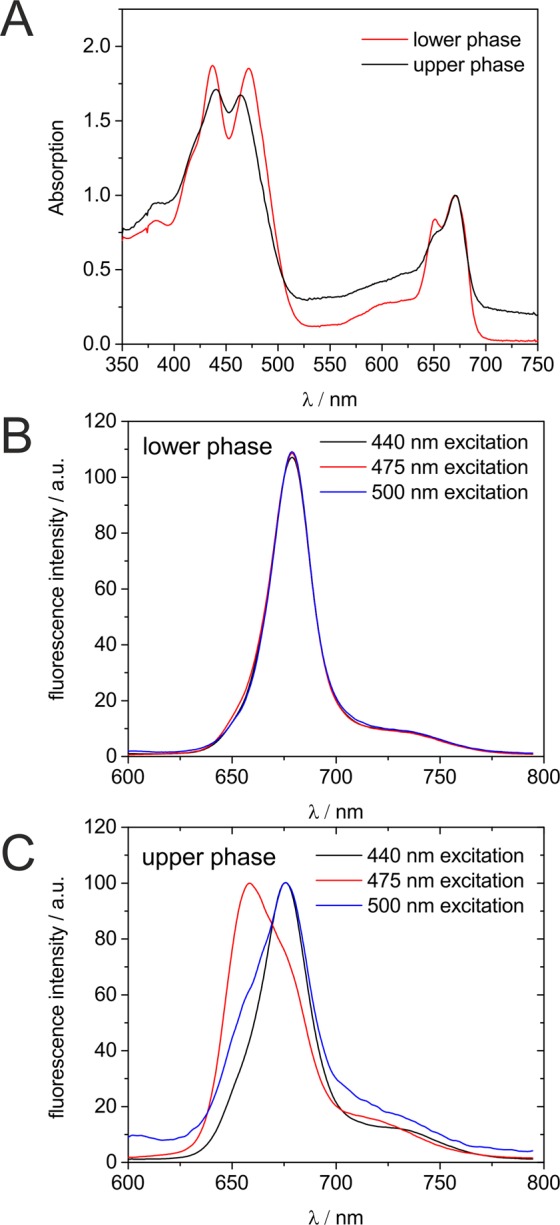
Spectroscopic analysis of sucrose gradient fractions containing refolded His-LHCBM6. (**A**) Absorbance spectra of lower and upper phase fractions in the range of 350 to 750 nm. (B + C) Fluorescence emission spectra of lower (**B**) and upper (**C**) phase fractions recorded with an excitation at 440, 475 and 500 nm.

Previous studies have already demonstrated, that physisorption of LHCII molecules onto titanium dioxide photoanodes leads to photocurrent enhancement, but these studies were based on the use of LHCII proteins isolated from complex biological sources, such as spinach leaves^8–11^. We wanted to investigate, whether recombinant LHCII molecules represent a promising alternative as a dye for the bio-sensitization of photoanodes. For the *in vitro* reconstitution of LHCII proteins (Fig. 1A), the *C. reinhardtii* LHCII isoform LHCBM6 (UniProtKB - A8J287) was cloned into the *E. coli* expression vector pQE80L to enable IPTG-inducible expression of the protein (Fig. 1B; left panel), which contained a 6xHis-tag fused to its N-terminus. The chloroplast transit sequence (amino acids 1–15 at the N-terminus) as predicted by Predalgo^13^ was excluded from the coding sequence used for expression in *E. coli* (Supplemental Fig. 1; HisL6). Since LHCBM6 is an integral membrane protein^1^ it formed inclusion bodies in *E. coli*, which were purified to serve as a starting material for *in vitro* reconstitution (Fig. 1B; right panel; IBs; Supplemental Figs 2 and 3). The workflow of the reconstitution/refolding procedure is shown in Fig. 1A. Insoluble and misfolded His-LHCBM6 present in inclusion bodies is solubilized and unfolded by boiling in the presence of detergent, while refolding is induced by exchanging the ionic detergent LDS with the mild non-ionic detergent Octyl β-D-glucopyranoside in the presence of pigments (chlorophylls *a*/*b* and carotenoids) isolated from spinach leaves (Supplemental Fig 4). Excess pigments are removed from the refolded sample by chromatography on a Ni-NTA resin, before pooled eluted fractions containing His-LHCBM6 are subjected to centrifugation through a sucrose gradient. Centrifugation through the sucrose gradient resulted in a separation of the Ni-NTA eluted protein into two discrete bands (Fig. 1C). In order to confirm the success of the refolding procedure, spectroscopic analyses of both gradient fractions were conducted. For both gradient fractions absorbance spectra were recorded and only the fraction with the higher sedimentation velocity clearly displayed the expected Qy peaks at 671.5 nm and 651 nm as well as the Soret transitions at 439 nm and 466 nm^12^. Comparison of the absorbance spectra indicated that the lower phase contains properly refolded LHCBM6 protein, which was further confirmed by recording fluorescence emission spectra of both fractions after excitation at 440 nm, 475 nm and 500 nm (Fig. 2B, C). A perfect overlap of emission spectra in the case of the higher density fraction (Fig. 2B) demonstrates an unperturbed excitation energy transfer between bound pigments and proper refolding, while this could not be observed for the lower density fraction (Fig. 2C). Therefore, it could be concluded that the refolding procedure yielded functional light-harvesting protein LHCBM6.

### Continuous illumination of LHCII-based DSSCs leads to a photoactivation and photocurrent enhancement

**Figure 3:**
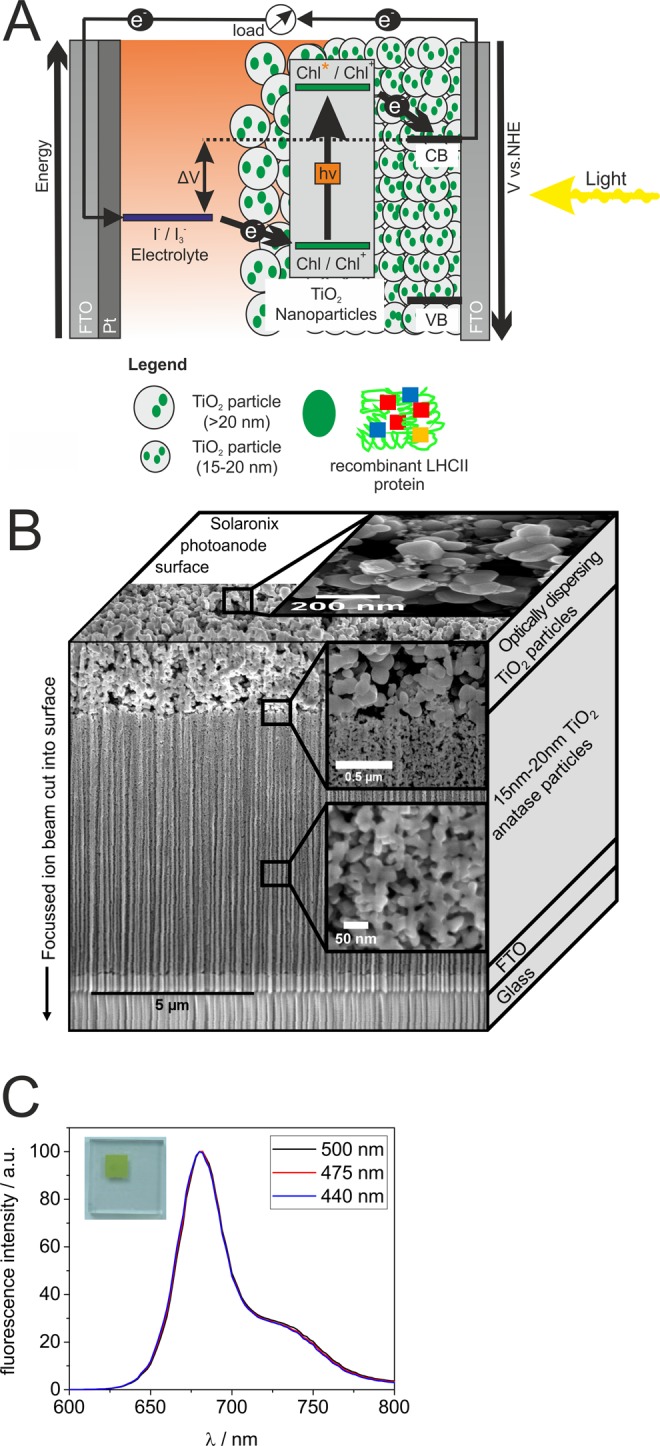
Composition of the DSSC used in the present work and fluorescence emission spectra of LHCII proteins physisorbed onto the photoanode. (**A**) Composition of the DSSC. Titanium dioxide nanoparticles (grey circles) are sensitized with light-harvesting proteins (green dots) containing chlorophyll pigments. Excited state chlorophylls inject electrons into the conduction band of titanium dioxide. Electrons are transferred to an FTO/platinum counter electrode at which iodine is reduced to iodide (I_3_^−^ + 2 e^−^ → 3 I^−^), while the reverse reaction (3 I^−^ → I_3_^−^ + 2 e^−^) fills the electron gap resulting from charge separation in the chlorophyll molecule. Two different layers of titanium dioxide are used with different sizes of titanium dioxide particles. (**B**) Composite scanning electron microscope picture showing the structure of the photoanode. A focused ion beam was used to cut into the surface to obtain depth information. The vertical line structure is a result of the ion beam cutting process. (**C**) Fluorescence emission spectra of His-LHCBM6 after physisorption onto titanium dioxide nanoparticles as part of the photoanode (photograph). Excitation wave lengths are indicated.

In order to construct a dye-sensitized solar cell (DSSC; Fig. 3A), refolded His-LHCBM6 protein was immobilized by physisorption on a Solaronix photoanode. The photoanode was composed of an indium tin oxide (InO_2_/SnO_2_) film, a mesoporous layer composed of titanium dioxide (anatase) nanoparticles with an average diameter of 15–20 nm and a macroporous layer containing particles with a larger diameter in order to improve light scattering^14^. It has already been shown in previous studies that LHCII proteins can be bound to titanium dioxide surfaces via the presence of carboxyl (−COOH) anchoring groups^9,10,15^ and His-LHCBM6 contains 14 glutamic acid and 11 aspartic acid residues for titanium coordination (Fig. S1A). Physisorbed His-LHCBM6 proteins retained their functional integrity as could be seen by recording fluorescence emission spectra following excitation at different wavelengths (Fig. 3B).

**Figure 4:**
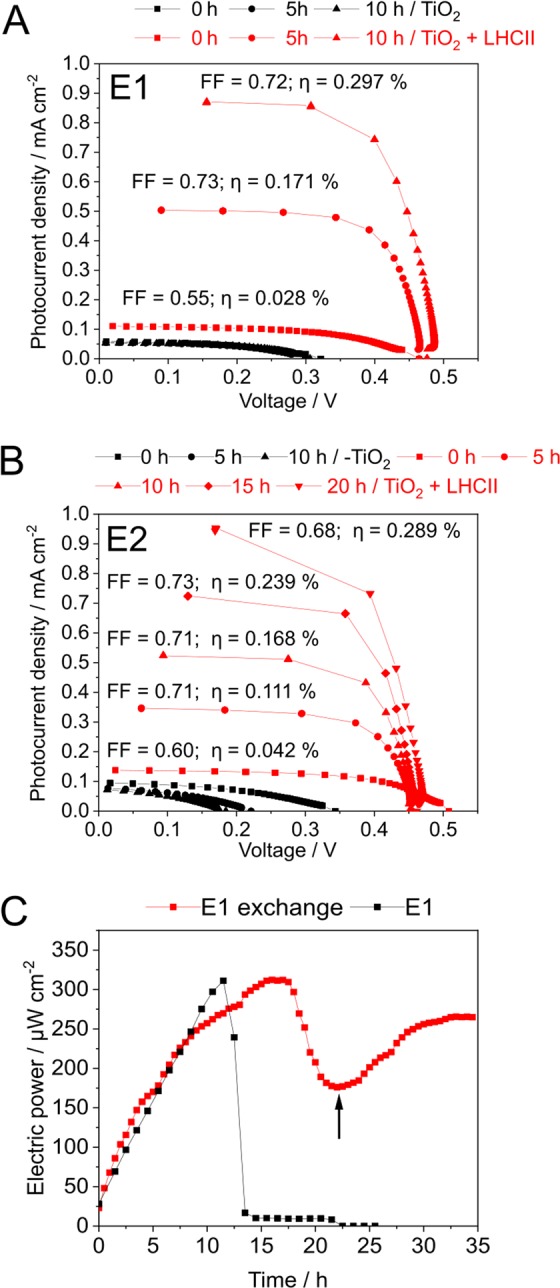
Correlation between illumination time and DSSC performance determined for two distinct electrolytes (**A**) *I*–*V* curves of LHCII-sensitized TiO_2_ solar cells in conjunction with an Iodolyte AN-50 (Solaronix) electrolyte (E1). Photoanodes without (TiO_2_; black curves) or with prior physisorption of LHCII proteins (TiO_2_ + LHCII; red curves) were continuously illuminated with 1 sun and using an AM 1.5 G filter. Photocurrent densities were determined directly after assembling the DSSC (t_0_) or after 5/10 h of illumination. *I–V* curves were used to calculate the solar energy to electricity conversion efficiency (η) as well as the fill factor (FF). (**B**) Measurements were conducted as in (A), but with electrolyte E2 (0.5 M LiI, 0.05 M I_2_, 0.3 M DMPII, 0.5 M 4-TBP)^8^. Additional photocurrent densities were recorded after 15 and 20 hours of illumination. (**C**) Maximum electric power (P_max_) generated during illumination. Several time points of illumination were analyzed for electrolyte E1. Measurements were either conducted without (black curve) or with an exchange of spent electrolyte against fresh electrolyte (red curve). A black arrow indicates the time point of electrolyte exchange.

*I–V* curves (Fig. 4) were then recorded in order to characterize the performance of DSSCs sensitized with recombinant LHCII proteins and illuminated with artificial sunlight, emitted by a xenon lamp. LHCII-sensitized photoanodes generated a much higher short circuit current per cm^2^ of electrode surface (Fig. 4A; red curves; TiO_2_ + LHCII) than bare titanium dioxide photoanodes (black curves; TiO_2_) and the performance of LHCII-containing photoanodes improved significantly during illumination.

An improved performance of LHCII-based DSSCs upon storage after assembly has been shown in previous studies^8,9^ and it was hypothesized that this is due to LHCII aggregation enabling the formation of additional Chl-Chl charge transfer states with improved electron injection into the conduction band of TiO_2_^9^. An activation of the DSSC during continuous illumination, however, is a novel finding, because previous published studies did not analyse the changes in DSSC performance within extended operation periods.

The solar energy to electricity conversion efficiency (η) increased about ten-fold from 0.028% at t_0_ to 0.297% after ten hours of illumination (Fig. 4A; 0 vs. 10 h; red curves). At this time point the photocurrent density of LHCII-sensitized photoanodes exceeded the respective current density of bare titanium dioxide by 15-fold (0.87 mA·cm^−2^ vs. 0.058 mA·cm^−2^). This increase in J_sc_ upon inclusion of LHCII molecules into the DSSC is unprecedented, since relative increases in J_SC_ reported by others were only in the range of 1.5–2-fold^9,10^. An activation of LHCII-sensitized photoanodes during illumination could also be noted, when another electrolyte (E2) containing 4-tert-butylpyridine and guanidinium thiocyanate (GuNCS) was used (Fig. 4B; red curves; 0–20 h). These additives are known to prevent recombination processes on the TiO_2_ surface, thus enhancing electron injection^8,16^. Again, physisorption of LHCII proteins dramatically increased the photocurrent density by ~10-fold compared to photoanodes composed of titanium dioxide alone. In the presence of additives contained in electrolyte E2 even higher photocurrent densities of 0.94 mA·cm^−2^ could be noted after reaching the maximal activation state of the photoanode (Fig. 4B; TiO_2_ +LHCII; 20 h).

Maximum photocurrent densities reached upon photoactivation of the DSSC were 0.94 mA·cm^−2^ (E2) and 0.87 mA·cm^−2^ (E1), respectively. This is in the range^8,10^ or far above the range^9,11^ of photocurrent densities obtained with LHCII-sensitized DSSCs reported before (equivalent of 1 sun; 100 mW·cm^−2^; AM 1.5 G filter applied). Our best solar energy to electricity conversion efficiencies (η ~ 0.3%) were comparable to those determined in other published studies (η ~ 0.5%^10^; η ~ 0.8%^8^). Conversion efficiencies obtained with LHCII-based bio-dyes were, however, far lower than those reported for Ruthenium-based organic dyes (η in the range of 3–11%)^17,18^, frequently used in conjunction with TiO_2_ photoanodes.

### DSSC deactivation is unrelated to a reduced dye performance

We repeated the photocurrent measurements with LHCII-based DSSCs in conjunction with electrolyte E1 in order to determine the end of the photoactivation phase, by analysing the maximum electric power (P_max_) generated during DSSC illumination over a longer period (Fig. 4C). An activation during the first 12 hours was followed by a sharp decline in P_max_ (12–25 h). In order to exclude that a denaturation or photobleaching of LHCII molecules during DSSC operation was responsible for the electric power decline observed in the short deactivation phase, an additional experiment with an exchange of spent electrolyte E1 with fresh electrolyte was performed (Fig. 4C; E1 exchange; black arrow). Addition of fresh electrolyte to the DSSC within the deactivation phase led to a recovery of the DSSC, as seen by a re-increase of the maximum electric power obtained. Therefore, an inactivation or desorption of light-harvesting proteins as the cause for DSSC activation can be ruled out, pointing at electrolyte (E1; Iodolyte AN-50 from Solaronix) decomposition as the cause for the decline in DSSC performance. Electrolyte bleaching, the disappearance of I_3_^−^ ions, has been proposed as a mechanisms of DSSC inactivation before^19^. The conversion of I_3_^−^ to iodate (IO_3_^−^) was proposed as the mechanism leading to electrolyte bleaching, which was in turn thought to be promoted by the inevitable presence of water in the assembled DSSC, raising the pH by activating pyridine base additives, thus reducing the stability of triiodide^7^. Since the E1 electrolyte used within the DSSC contains a pyridine additive, electrolyte bleaching might have caused the observed loss in DSSC activity during operation. Importantly, however, the fact that photocurrent generation can be restored by adding fresh electrolyte excludes LHCII desorption and denaturation of LHCII proteins as the main cause of the observed phenomenon.

### Long-lasting photocurrent production for several days with LHCII-sensitized DSSCs and UV-depleted light

**Figure 5:**
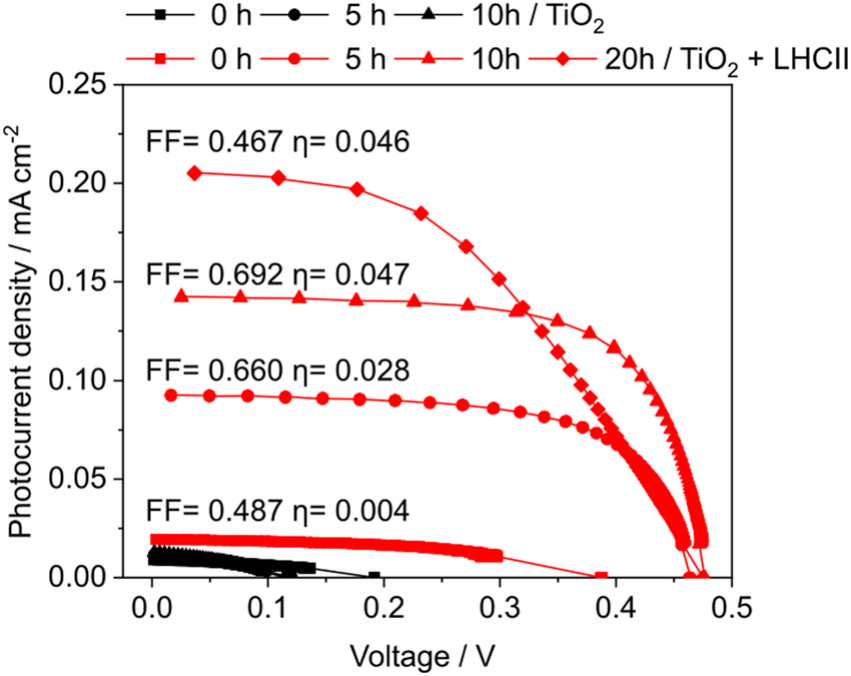
DSSC performance under illumination with light lacking the UV part. *I*–*V* curves of LHCII-sensitized TiO_2_ solar cells in conjunction with an Iodolyte AN-50 electrolyte (E1). Photoanodes without (TiO_2_) or with prior physisorption of LHCII proteins (TiO_2_ + LHCII). Photocurrent densities were determined directly after assembling the DSSC (t_0_) or after 5 and 10 h of illumination. *I–V* curves were used to calculate the solar energy to electricity conversion efficiency (η) as well as the fill factor (FF).

**Figure 6:**
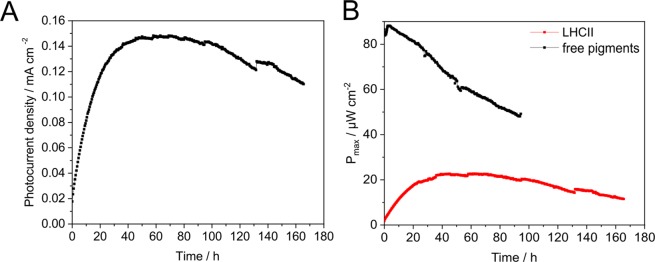
LHCII-sensitized TiO_2_ photoanodes maintain a high photocurrent during prolonged illumination times. (**A**) Maximum photocurrent densities determined during a prolonged operation period of LHCII-sensitized titanium dioxide photoanodes. (**B**) The maximum electric power generated during DSSC operation over time. LHCII-sensitized photoanodes (LHCII; red curve) were compared to photoanodes sensitized with free photosynthetic pigments (chlorophylls and carotenoids) isolated from spinach leaves (free pigments; black curve).

The photosynthetic pigments (chlorophylls a/b and carotenoids) bound to LHCII molecules show their strongest absorbance in the 400 to 700 nm range, the so-called PAR (photosynthetically active radiation) range^3^. We therefore repeated photocurrent measurements by applying a UV filter, in order to exclude radiation with a wavelength below 400 nm from the light for DSSC irradiation (Fig. 5). Again, we could observe an increase in J_SC_ during DSSC operation, when TiO_2_ was sensitized with LHCII molecules (Fig. 5; TiO_2_ + LHCII; 0 vs. 20 h). Compared to measurements conducted with the full artificial sunlight spectrum (Fig. 4), however, the maximum J_SC_ obtained with UV-depleted light was about five times lower (~0.2 mA·cm^−2^ vs. ~1 mA·cm^−2^ (+UV)) and η around six times lower (0.289–0.297% vs. 0.046%), which can be explained by the diminished contribution of charge separation in the large band gap semiconductor TiO_2_ to photocurrent generation, when UV light is excluded^20^. *I*–*V* measurements were repeated with an extended operation time of the DSSC in order to determine the length of the activation and deactivation phase (Fig. 6A). Within the first 30 h a sharp increase in J_SC_ was notable, which was followed by a slow deactivation beyond this time point up to the seventh day (t = 170 h). When free photosynthetic pigments were physisorbed onto the photoanode and P_max_ measured in order to asses DSSC performance, an opposing trend was noted (Fig. 6B; free pigment vs. LHCII). While LHCII-sensitized DSSCs showed an activation phase followed by a slow decrease in P_max_, the electric power generated by pigment-sensitized DSSCs displayed a steady decline, indicating an overall lower stability of the device. Chlorophylls, not bound to light-harvesting apoproteins, rapidly bleach under illumination, since singlet oxygen scavenging and quenching of chlorophyll triplets, facilitated by xanthophylls located in close proximity within LHCII molecules^1^, are not present. A higher P_max_ of the pigment-sensitized DSSC in the beginning of the measurements can be explained by a higher dye density.

Overall, we present data demonstrating the suitability of recombinant LHCII proteins from the green microalga *C. reinhardtii* as a dye in bio-sensitized solar cells. In contrast to previous studies, long-term measurements of DSSC performance under continuous illumination and an operation of the device for several days were conducted (Fig. 6A). These long-term analyses revealed that during continuous illumination an activation of the DSSC occurs, which is reversed in a second phase. We currently cannot depict the mechanisms underlying the impressive rise in photocurrent densities observed within the activation phase (Figs 4A, B and 5), but it seems reasonable to suggest that a re-organization of LHCII molecules on the TiO_2_ nanoparticle surface is one of the contributing mechanisms. The aggregation state of LHCII molecules physisorbed onto titanium dioxide surfaces has already been shown to have a great impact on photocurrent generation, and was explained by the promotion of Chl-Chl charge transfer states, facilitating electron injection into the semiconductor^9^. An exchange of the electrolyte (Fig. 4C) excluded an inactivation of the LHCII bio-dye as the cause for a decline of photocurrents in the deactivation phase. The precise mechanism of electrolyte decomposition is difficult to explain, considered that although several hypotheses regarding the detrimental role of trace amounts of water inevitably present in DSSCs were formulated, the actual consequence of water contamination in DSSCs remains enigmatic^7^. In fact, a study published by O’Regan and coworkers in 2010 and showing an unaffected J_SC_ up to the presence of 40% (v/v) in the electrolyte^21^, lead to a novel view on water as DSSC component^7^. Independent of the actual mechanism which interferes with electrolyte function, a higher electron turnover in the presence of UV light seems to accelerate the process, as can be seen by comparing the duration of the activation phase in measurements conducted with (Fig. 4) and without (Fig. 6) UV light. The use of heterologous expression in *E. coli* together with refolding opens up novel opportunities to create bespoke bio-dyes based on light-harvesting proteins. For instance, replacing Chl *a* by Chl *f*/*d* during the refolding process might be a means to extend the PAR region via red-shifted chlorophylls^3^. The process applied to generate LHCII bio-dyes in the present study should probably not be easily scalable and economically viable. Furthermore it cannot be considered sustainable since heterotrophic microorganisms and hence reduced carbon sources are used for cultivation. In addition organic solvents are applied to extract photosynthetic pigments from spinach leaves. Future strategies should therefore consider the production of tagged LHCII molecules directly in green microalgae from carbon dioxide in conjunction with simple purification methods, maybe based on direct absorption of LHCII molecules from crude extracts onto photoanodes.

## Methods

### Fabrication of DSSCs

Photoanodes (Solaronix Titania Electrodes, opaque, #74101) and cathodes (Solaronix Platinum Electrodes, drilled, #74201) were heated up from RT (~20 min.) and then baked at 450 °C for 30 min. (anode) or 10 min. (cathode). Cathodes were cooled down to RT, anodes cooled down to approx. 100 °C and put in a desiccator and vacuumed. The anodes were then transported to the bio lab for sensitization. After sensitization, the anode is covered by a mask (Solaronix #7401) to confine the radiation to an area slightly larger than the active area to obtain unbiased PV measurements. A gasket cut from Parafilm M (Bemis Company, Inc.) is put between the electrodes before clamping the electrodes with common foldback clips. Electrolyte was then filled by a special syringe trough a hole in the cathode (Solaronix Vac‘n’Fill Syringe). The hole was sealed by adhesive tape. Square-shaped photoanodes had an active area of 36 mm² (6 mm · 6 mm).

### Scanning electron microscopy

The focused ion beam of a FEI Helios NanoLab 600 DualBeam (FIB/SEM) was used to cut at an angle into the surface of a baked and cooled down (see Fabrication of DSSCs) photoanode. Using the electron beam, images were taken of the surfaces with varying magnifications.

### Characterization of DSSCs

A custom build solar simulator with a xenon lamp (Ushio UXL-150SO), an AM1.5 G filter (Newport Spectra-Physics #81094) and an optional UV filter (Solaronix #49132) was used to illuminate the DSSC. The electrical measurement is performed by varying a programmable resistor decade (ELV EWD100) and measuring voltage and current with two multimeters (Uni-T UT804) which transfer the data to a computer for analysis and storage.

### Preparation Lhcbm6 apoprotein

*LHCBM6* cDNA from *Chlamydomonas reinhardtii* was cloned into the pQE80L expression vector (Qiagen) containing an N-terminal 6xHis-tag. The codon-optimized *LHCBM6* gene was transformed into *E. coli* BL21(DE3) and cells were cultivated in LB-media^22^ at 37 °C until reaching an OD_600_ of 0.6. Protein expression was induced by 1 mM isopropyl-β-D-thiogalactopyranosid (IPTG) for 4 h. Cells were harvested by centrifugation for 5 min. at 5000 × g and 4 °C. The pellet was treated with BugBuster reagent (Novagen) and the inclusion body preparation performed according to the manufacturer’s instruction.

### Pigment isolation and reconstitution of LHCBM6-pigment complexes

Total pigment extract was isolated from fresh spinach by acetone extraction. Spinach leaves were grinded in buffer containing 25 mM Tris-HCl pH 7.8, 1 mM dithiolthreitol (DTT) and 330 mM sorbitol at 4 °C. The suspension was filtered through three layers of Miracloth (Calbiochem) and centrifuged for 10 min. at 8000 × g and 4 °C. The pellet was resuspended in cold aceton (containing NaCO_3_) and the centrifugation repeated. Pigments from the supernatant were extracted with diethylether and the solvent vaporized prior to the storage of pigments at −80 °C. The *in vitro* reconstitution method is described in Giuffra *et al*.^23^.

### Purification of reconstituted Lhcbm6

To remove excess pigments and misfolded protein a Ni-NTA affinity chromatography was performed. Ni-NTA-resin (Qiagen) was washed with buffer containing 1% (v/v) octyl β-D-glucopyranoside (OGP), 12,5% (w/v) sucrose, 200 mM NaCl, 10 mM imidazol, 20 mM Hepes pH 7.6. The reconstituted LHCBM6-(His)_6_ was eluted with buffer containing 250 mM imidazol, 200 mM NaCl, 0.06% (w/v) n-dodecyl β-D-maltoside (β-DM), 40 mM Hepes pH 7.6.

### Spectroscopy

Absorption spectra were recorded at room temperature using Genesys 10 S UV-Vis spectrometer (Thermo Scientific) and VisionLite software version 4.0. The spectra were recorded from 350 to 750 nm in 1 nm intervals. Chlorophyll content was determined by measuring the absorbance at 645 nm and 663 nm and calculated according to Porra *et al*.^24^. Fluorescence analysis of reconstituted LHCBM6 in sucrose buffer and of TiO_2_/ITO plates with and without proteins were performed with Tecan infinite M200 Reader in 96-well plates and 6-well plates, respectively. Spectra were measured with excitation wavelength 440 nm, 475 nm and 500 nm. Emission was recorded from 600 nm to 800 nm in 2 nm intervals.

### Coating of LHC on electrodes

Incubation of TiO_2_/ITO electrodes occurred in a saturated LHCBM6 solution for 16 h at 4 °C in dark. Electrodes were rinsed with distilled water to remove excess proteins and stored in the dark at 4 °C.

## Supplementary information

Supplementary Information
