# Supplementary material for: Extremely robust photocurrent generation of titanium dioxide photoanodes bio-sensitized with recombinant microalgal light-harvesting proteins
Source: Sci Rep. 2019 Feb 14;9:2109. doi: 10.1038/s41598-019-39344-6 (PMC6376048; doi:10.1038/s41598-019-39344-6)
Supplement: Supplementary file 1 — Supplementary Information [file 41598_2019_39344_MOESM1_ESM.pdf]

Supplementary Information

Extremely robust photocurrent generation of titanium dioxide photoanodes bio-sensitized with recombinant microalgal light-harvesting proteins

Nina Lämmermann[1], Fabian Schmid-Michels[2], Aike Weißmann[2], Lutz Wobbe[1], Andreas Hütten[2*] & Olaf Kruse[1*]

[1]Bielefeld University, Faculty of Biology, Center for Biotechnology (CeBiTec), Universitätsstrasse 27, 33615, Bielefeld, Germany
[2]Bielefeld University, Department of Physics, Center for Spinelectronic Materials and Devices, Universitätsstrasse 25, 33615 Bielefeld
Corresponding authors: *olaf.kruse@uni-bielefeld.de; *andreas.huetten@uni-bielefeld.de

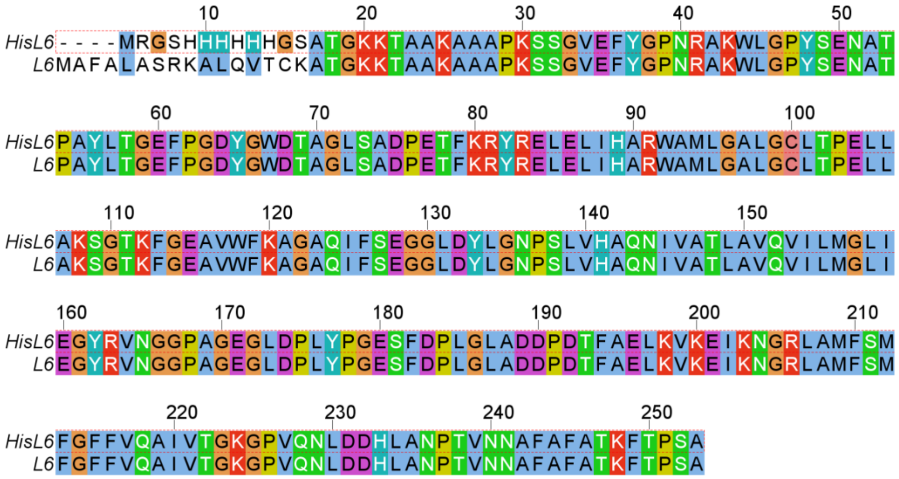

Supplemental Figure 1. Amino acid sequence alignment of LHCBM6 from *Chlamydomonas reinhardtii* (UniProtKB - A8J287; L6) and His-L6, the sequence used for heterologous expression in *E. coli*.

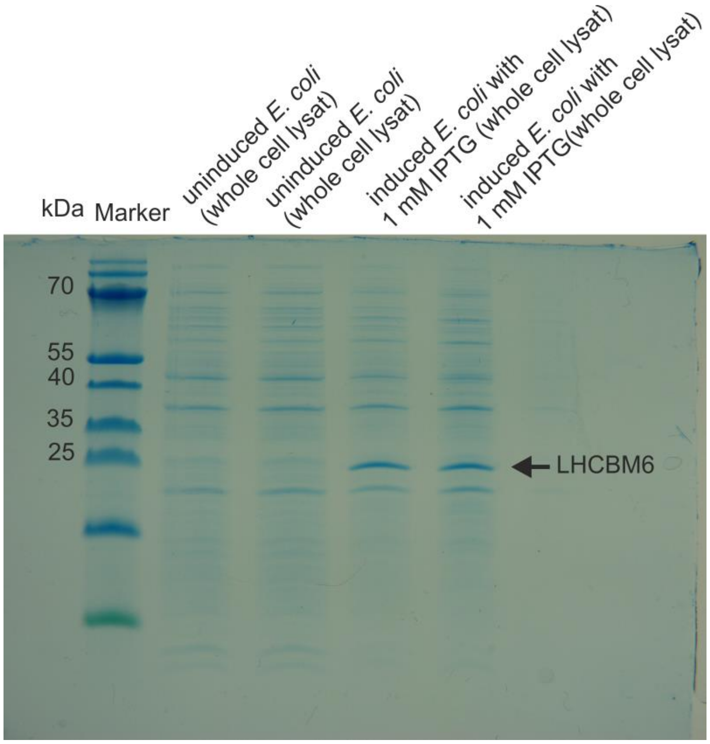

Supplemental Figure 2. Coomassie Brilliant Blue-stained SDS-PAGE (12%) gel. Whole cell lysates from *E. coli* cultures harbouring the expression construct for His-LHCBM6 were separated after cultivation in the absence (uninduced) or presence of 1 mM IPTG as an inductor (induced). An arrow indicates the position of His-LHCBM6.

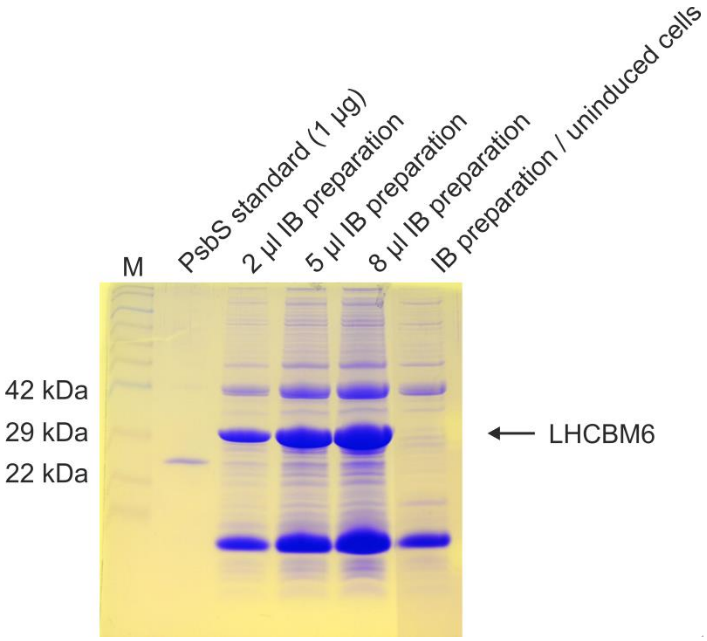

Supplemental Figure 3. Coomassie Brilliant Blue-stained SDS-PAGE (12%) gel. Inclusion body (IB) preparations from *E. coli* cultures harbouring the expression construct for His-LHCBM6 were separated after cultivation in the absence (uninduced) or presence of 1 mM IPTG as an inductor. An arrow indicates the position of His-LHCBM6.

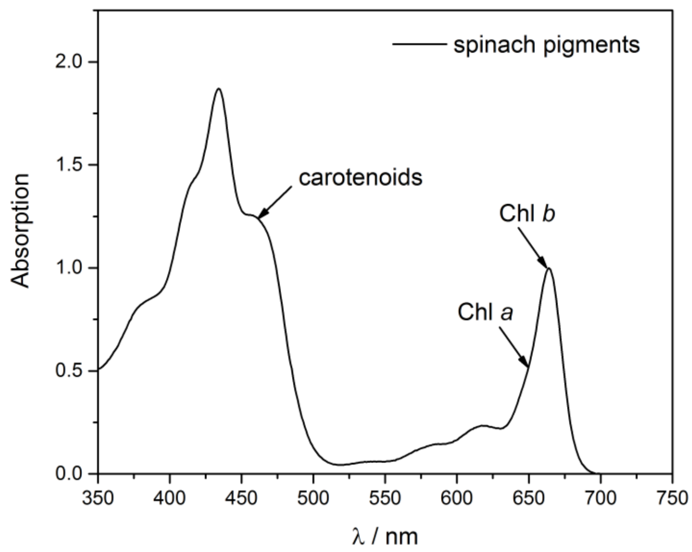

Supplemental Figure 4. Absorption spectrum of a pigment preparation isolated from spinach leaves. The position of absorbance bands indicating carotenoids, chlorophyll a (Chl a) and chlorophyll b (Chl b) is marked by arrows.
